# Mutation in DNA Polymerase Beta Causes Spontaneous Chromosomal Instability and Inflammation-Associated Carcinogenesis in Mice

**DOI:** 10.3390/cancers11081160

**Published:** 2019-08-13

**Authors:** Shengyuan Zhao, Alex W. Klattenhoff, Megha Thakur, Manu Sebastian, Dawit Kidane

**Affiliations:** 1 Division of Pharmacology and Toxicology, College of Pharmacy, Dell Pediatric Research Institute, The University of Texas at Austin, 1400 Barbara Jordan Blvd. R1800, Austin, TX 78723, USA; 2 Department of Epigenetics and Molecular Carcinogenesis, The University of Texas MD Anderson Cancer Center, Science Park, Smithville, TX 78957, USA

**Keywords:** DNA polymerase beta, mutation, genomic instability, inflammation

## Abstract

DNA polymerase beta (Pol β) is a key enzyme in the base excision repair (BER) pathway. Pol β is mutated in approximately 40% of human tumors in small-scale studies. The 5´-deoxyribose-5-phosphate (dRP) lyase domain of Pol β is responsible for DNA end tailoring to remove the 5’ phosphate group. We previously reported that the dRP lyase activity of Pol β is critical to maintain DNA replication fork stability and prevent cellular transformation. In this study, we tested the hypothesis that the human gastric cancer associated variant of Pol β (L22P) has the ability to promote spontaneous chromosomal instability and carcinogenesis in mice. We constructed a Pol β L22P conditional knock-in mouse model and found that L22P enhances hyperproliferation and DNA double strand breaks (DSBs) in stomach cells. Moreover, mouse embryonic fibroblasts (MEFs) derived from L22P mice frequently induce abnormal numbers of chromosomes and centrosome amplification, leading to chromosome segregation errors. Importantly, L22P mice exhibit chronic inflammation accompanied by stomach tumors. These data demonstrate that the human cancer-associated variant of Pol β can contribute to chromosomal instability and cancer development.

## 1. Introduction

Base excision repair (BER) is the predominant pathway in cells for preventing mutations associated with oxidative and alkylating DNA damage [1,2,3,4]. BER is initiated by excision of damaged bases by DNA glycosylases which generate an abasic site. Subsequent steps may follow either of two distinct BER subpathways that replace either a single nucleotide (short-patch BER) or multiple nucleotides (long-patch BER). Repair of an abasic site is initiated by AP endonuclease 1 (APE1), which generates a nick between the 5´-deoxyribose-5-phosphate (5’-dRP) and the 3’ hydroxyl group [5]. Following this incision of DNA, the 8 kDa dRP lyase domain of DNA polymerase beta (Pol β) functions by removing the 5’-dRP group, and the 31 kDa polymerase domain of Pol β then fills the gap. Pol β is the key enzyme in mammals for repairing DNA lesions resulting from oxidation or alkylation via its role in short patch BER [6,7]. The major cellular roles of Pol β include removal of the 5′-dRP group from the end of the DNA strand and filling the gap during BER [8,9,10,11]. The dRP lyase activity of Pol β is required to remove 5′-dRP group residues in mammalian cells [6,10]. Mutations in the Pol β gene that lead to inefficient 5′-dRP removal likely result in accumulation of BER intermediates that lead to genomic instability. To allow gap filling by Pol β and rejoining by DNA ligase, these unconventional ends have to be restored to the conventional 3′-OH and 5′ phosphate ends. The 5′-dRP moiety generated by the AP endonuclease can be removed by the 5′-dRPase activity of Pol β [10]. Several studies show that Pol β overexpression induces genetic instability in mammalian cells by triggering an alternative DNA repair pathway [12]. In contrast, Pol β deficiency is associated with the accumulation of single strand breaks (SSBs) [13]. Unrepaired SSBs can convert into double strand breaks (DSBs) in replicating cells due to the collapse of the DNA replication fork [14]. Mice deficient in Pol β die immediately after birth with high levels of apoptosis of the newly generated postmitotic neurons [15], although immortalized mouse embryonic fibroblasts (MEFs) lacking Pol β are viable [6].

Pol β is mutated in 40% of human tumors in small-scale studies and likely drives tumorigenesis through genomic instability [16,17]. Eight percent of Pol β mutations occur in the dRP lyase domain [17,18]. Mutation in the polymerase domain of Pol β or complete deletion of the Pol β gene causes genomic instability in mitotic and meiotic cells, respectively [19,20]. The dRP lyase function of Pol β plays a more significant role in removal of the 5’-dRP group than any other DNA polymerase [21,22] and protects cells from DNA damage-induced cytotoxicity [7]. L22P was discovered as a gastric cancer-associated heterozygous variant of Pol β [23] and was found to lack dRP lyase activity and DNA-binding affinity in vitro [24].

Our previously published data have shown that cells with the L22P mutation accumulate DSBs in the gastric epithelium and undergo cellular transformation [25]. However, there are no data regarding how dRP lyase-deficient Pol β contributes to chromosomal instability and promotes gastric carcinogenesis in a mouse model. Thus, we hypothesize that the L22P mutation of Pol β causes chromosomal segregation defects and promotes inflammation associated gastric carcinogenesis. We have constructed an L22P conditional knock-in mouse model at the *ROSA26* locus using the *Cre-loxP* targeting system to uncover the dominant negative consequence of aberrant BER-associated chromosomal instability and carcinogenesis in mice. 

In the current study, we report that the L22P mutation induces BER intermediates and replication-associated DSBs. Cells with unrepaired DSBs likely progress to the mitotic stage of the cell cycle and undergo chromosome segregation defects, including anaphase bridge-mediated micronuclei [26,27]. Moreover, we found centrosome amplification and cytokinesis failure in L22P cells. Thus, our findings demonstrate that the dRP lyase function is required to suppress mitotic defects. Furthermore, to clarify the role of Pol β in tumor initiation and progression, we examined whether L22P mice that are predisposed to chronic inflammation and ultimately develop cancer. Overall, our data suggest that Pol β is required to sequester chromosomal instability and inflammatory response to suppress tumor development. 

## 2. Results

### 2.1. Generation of L22P Mutant Mice

**Figure 1:**
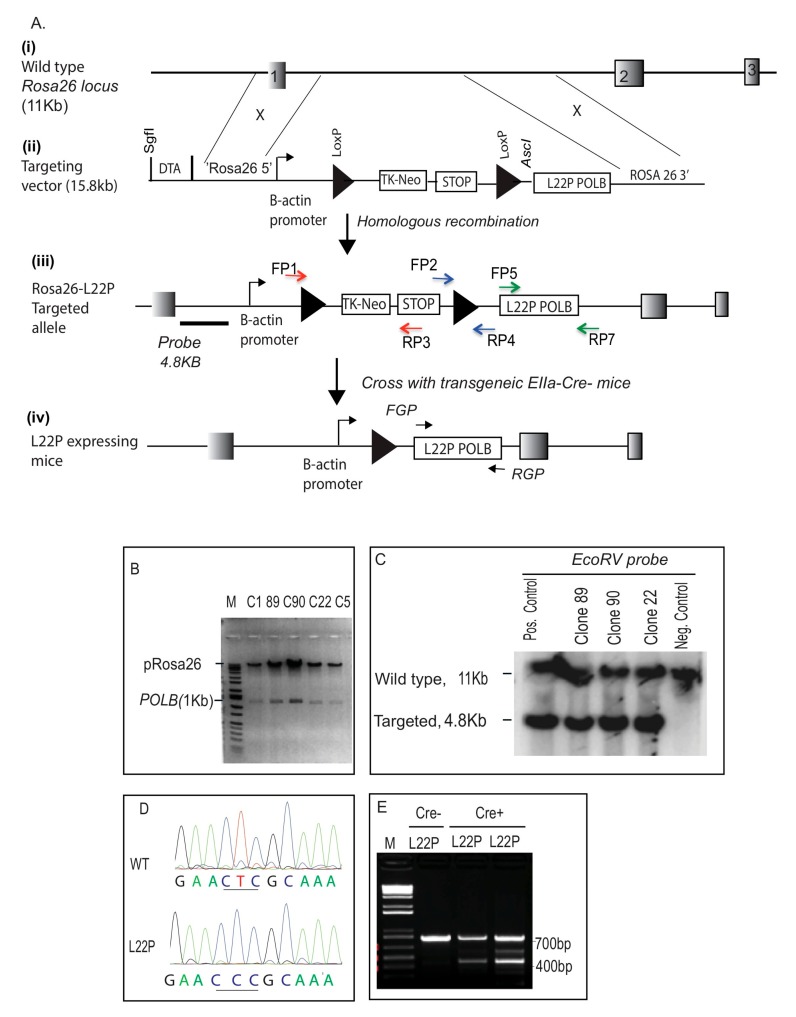
Conditional targeted knock-in mice at the *ROSA26* locus. (**A**) Schematic of the construction of the conditional L22P floxed allele. (i) *ROSA26* locus at chromosome 6 indicated as Exon 1, 2, and 3; (ii) Targeting construct of *POLB*; (iii) Targeted construct integrated at *ROSA26* locus via homologous recombination; Targeted alleles in embryonic stem (ES) cells were genotyped using allele-specific PCR at three different regions of the construct using different primer sets that are indicated with red, blue, and green (all primers listed in Table S1); Linear plasmid digested with *SgfI* was electroporated into ES cells derived from the B6/129SvJ strain; Neomycin genes flanked by the loxP site and diphtheria toxin (DTA) were used for positive and negative selection, respectively. (iv) Breeding strategy of constructed L22P mice with Cre-expressing mice to generate L22P-expressing mice; (**B**) Cloning of the wild-type *POLB* gene into the *AscI/XmaI* site of a modified plasmid carrying loxp-STOP-loxp. The positive clone contains a Pol β gene insert (1 kB), and the 15 kB fragment is all loxP and DTA at *ROSA26* loci and neomycine selection cassettes; (**C**) ES cells that were positive in PCR were confirmed using Southern blot hybridization. Southern blot analysis was performed on *EcoRV* digested genomic DNA extracted from the ES clones. Southern blotting reconfirms the integration of L22P Pol β at the *ROSA26* locus; clones ES89, 90, and 22 are positive for the 4.8 kb integration size; (**D**) Sequencing of the cDNA amplified Pol β gene from L22P mice in order to reconfirm the codon of leucine (CTC) changes to proline (CCC); (**E**) ES clone 89 was used to generate mice and the F1 generation was crossed with EIIa-Cre mice (constitutive Cre). Mice were genotyped for the Cre transgene, L22P allele at the *ROSA26* locus.

To test our hypothesis that L22P mutations lead to cancer, we have constructed mice that express an L22P variant using standard gene-targeting methods [28]. As shown in Figure 1A, we engineered a gene-targeting construct containing the L22P variant as a dominant negative mouse model. We inserted this construct into the *ROSA26* locus downstream of a stop signal flanked by two *LoxP* cassettes in targeting construct (Figure 1A (i and ii) and B). This design ensured that L22P could not be expressed until breeding with a Cre-recombinase-expressing mouse was performed. The targeting construct was linearized with *SgfI* restriction enzyme and electroporated into embryonic stem (ES) cells by the Transgenic Mouse Facility (Yale University). Two hundred ES cell clones were selected in neomycine and diphtheria toxin (DTA); this process ensured that recombination occurred before the thymidine kinase (*tk*) gene and was therefore targeted to the *ROSA26* locus (Figure 1A (iii)). DNA was isolated and amplified with primers that were designed to amplify a fragment that is integrated via homologous recombination (Figure 1A (iii); blue, red and green arrows). Positive clones for the targeted construct (clones, 22, 89 and 90) were subjected to Southern blotting using a probe that recognizes the *ROSA26-POLB* construct to ensure that the identified clones carry only targeted recombinants (Figure 1C). The results in Figure 1C show that clones 22, 89, and 90 appear to have targeted alleles of the *POLB* locus. As expected, clones that were not identified in the PCR assay did not possess a targeted allele (negative control). The presence of L22P mutation of *POLB* gene was confirmed by sequencing as shown in Figure 1D. The ES cells (from clone#89) were microinjected into C57Bl/6 blastocysts and implanted into pseudopregnant females. Chimeras exhibiting a high level of agouti coat color were born and mated to WT C57Bl/6 mice to produce F1 L22P offspring and genotyped using primers to amplify STOP signal plus downstream sequence that contain the L22P *POLB* gene (700 bp). The F1 L22P offspring were bred with EIIa-Cre mice and further genotyped with PCR using primers to confirm that the STOP signal was removed from upstream of L22P as indicated with the amplified 400 bp from Cre+L22P (cyan and yellow arrows, Figure 1 (iv) and PCR product on Figure 1E). In contrast, the PCR product that shows only 700 bp indicates Cre^−^ L22P mice.

### 2.2. L22P Increases Cellular Proliferation in Stomach Cells

**Figure 2:**
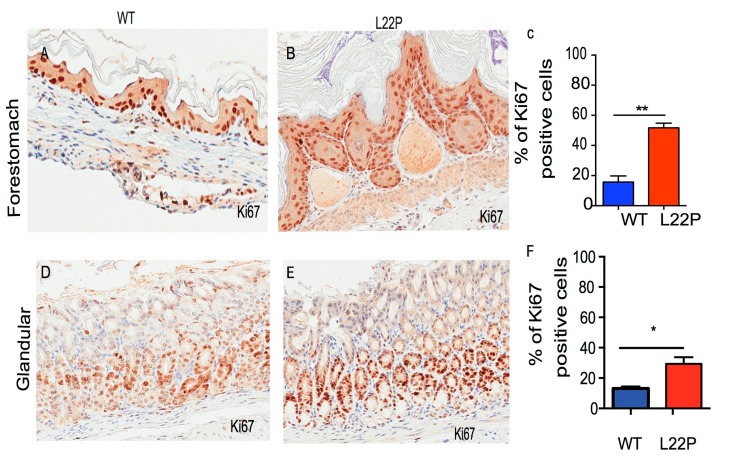
L22P mutation induces cellular proliferation in mice. (**A**,**B**) Representative image of forestomach tissue sectioned from wild type (WT) and L22P mice stained with Ki67 antibody proliferation markers; (**C**) Percent of cells positive for the Ki67 proliferation marker in forestomach of WT (*n* = 2400) and L22P (*n* = 2962) cells from forestomach. (**D**,**E**) Representative image of glandular stomach tissue sections from WT and L22P mice stained with Ki67; (**F**) Percent of Ki67 positive cells in WT (*n* = 6240) and L22P (*n* = 7750); Note that three mice from each group of WT and L22P genotypes were included to estimate the difference. The Mann–Whitney test was also applied to compare differences in Ki67 and statistical significance is designated by * *p* < 0.05; ** *p* < 0.01. All statistical analyses were performed using GraphPad Prism (Prism 7.00 for Mac, GraphPad Software, San Diego, CA, USA). All images shows 10× magnification and were captured using Scanscope (Leica Biosystem, Vista, CA, USA).

To determine whether L22P mutation alters cellular proliferation, we examined the homeostasis of proliferation in L22P and wild type (WT) mouse stomach tissues sectioned from eight-week-old mice and stained with Ki67 antibody. Ki67 is used to identify the proliferating cell compartment in stomach tissue. We found that the number of Ki67-positive cells was significantly increased in L22P versus WT mouse in the forestomach and glandular section of stomach tissues (Figure 2A–F; *p* < 0.01 and *p* < 0.05). Next, we asked whether there are any differences in apoptotic response induced by L22P as compared with WT mice stomach cells. We stained the stomach tissues with cleaved caspase-3 antibodies (Figure S1A–H). We found no statistically significant difference between the number of apoptotic cells in forestomach tissue of WT and L22P mice (Figure S1A–C). In contrast, the number of apoptotic cells moderately increased in L22P glandular stomach tissues versus WT (Figure S1D–F). This data demonstrates that normal dRP lyase function of Pol β is likely required to suppress proliferation of cells with unstable genomes in the stomach. 

### 2.3. L22P Induces Significantly Increased BER Intermidates and DSBs

**Figure 3:**
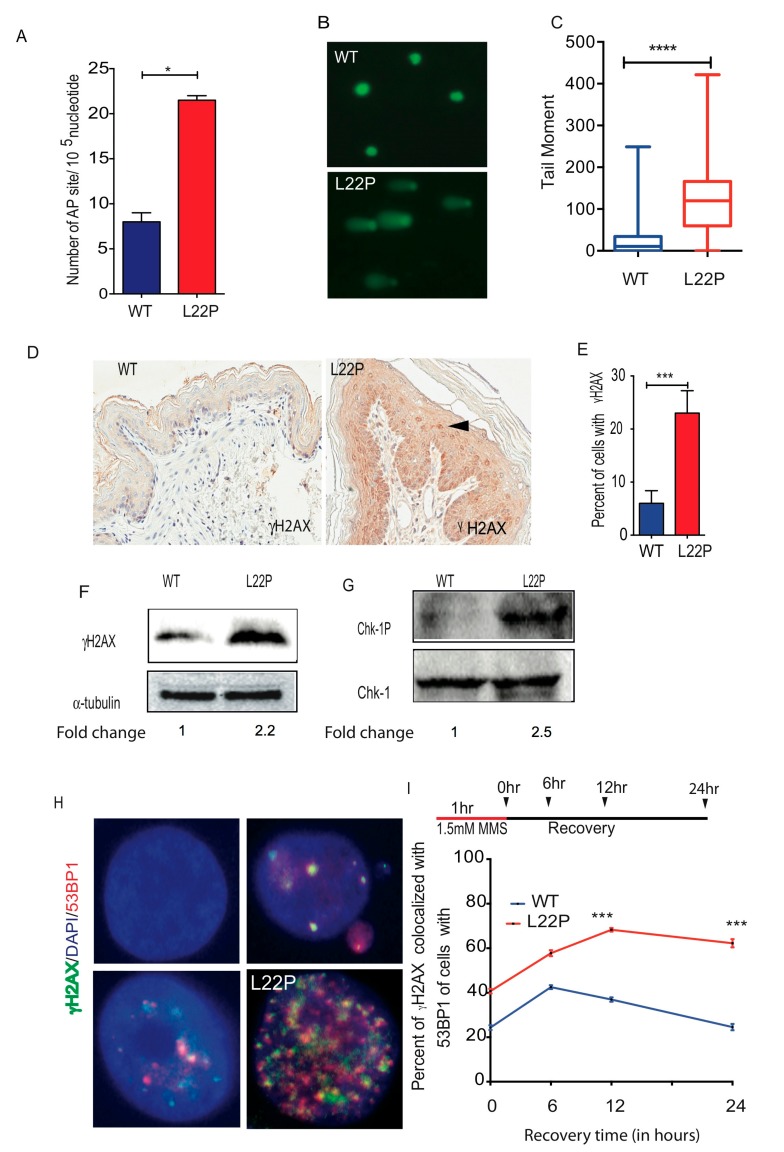
L22P mice accumulate base excision repair (BER) intermediates and exhibit double strand breaks (DSBs). (**A**) Number of apurinic/apyrimidinic (AP) sites/10^5^ nucleotides from DNA extracted from wild type (WT) and L22P mice stomach tissues; (**B**) Representative image of spontaneous single strand breaks (SSBs) from Alkali comet assay on WT and L22P mouse embryonic fibroblasts (MEFs) cells, All image captured with 63× objective of Zeiss fluorescence microscope (Zeiss, San Diego, CA, USA); (**C**) Measured tail moment for measuring spontaneous SSBs in WT and L22P MEFs cells and data analysis carried out using Student’s *t* test; (**D**) Immunohistochemical staining with phosphorylated H2AX (γH2AX) antibody of stomach tissue sectioned from WT and L22P mice; Image shows 5× magnification and were captured using Scanscope (Leica Biosystem, Vista, CA, USA); (**E**) Percent of γH2AX-positive cells in the WT (*n* = 2000) and L22P (*n* = 2078) mouse stomachs. Data were analyzed using Mann–Whitney test; (**F**) Western blot analysis of γH2AX in WT and L22P cells; Fold change calculated using the ratio of γH2AX band intensity to loading control of alpha-tubulin and normalized to wild type; (**G**) Western blot analysis for Chk1 and Chk1 phosphorylation at Ser 317; Fold change calculated using the ratio of band intensity of Chk1p to chk1 and normalized to wild type. (**H**) Representative image of the γH2AX and 53BP1 colocalization in WT and L22P MEFs; (**I**) Percent of cells showing colocalization of γH2AX and 53BP1 with Methyl methanesulfonate (MMS) DNA damage and recovery after treatment. L22P and WT MEFs were treated with 1.5 mM MMS and examined for the recovery of DSBs after removing MMS at the following times: 0 hours (WT *n* = 85; L22P *n* = 147); 6 hours (WT *n* = 106 versus L22P *n* = 108); 12 hours (WT *n* = 89 versus L22P *n* = 168); and 24 hours (WT *n* = 95 versus L22P *n* = 101). Note that all images were taken at 63× with a Zeiss fluorescence microscope (Zeiss, San Diego, CA, USA) with a constant exposure time, and any cell with >5 foci of γH2AXcolocalize with 53BP1/cell was categorized as a positive. Data were analyzed using two-way ANOVA test. Significant differences are designated by *** *p* < 0.001; **** *p* < 0.0001 using GraphPad Prism (Prism 7.00 for Mac, GraphPad Software, San Diego, CA, USA).

To determine whether L22P-expressing mice exhibit increased BER intermediates such as abasic sites (AP sites), we isolated DNA from stomach tissues using a DNAzole isolation kit and measured the number of AP sites using an AP site assay kit (Colorimetric; Abcam, Cambridge, MA USA). We found that cells from L22P mice had, on average, 22 AP sites/10^5^ nucleotides compared with ~9 AP sites/10^5^ nucleotides in cells from WT mice (Figure 3A). Furthermore, those processed AP sites likely induce single strand breaks (SSBs) in cells from L22P mice that are unable to repair. To determine whether L22P AP sites accumulate single strand breaks, we performed Alkali comet assays and found that SSBs significantly increase in L22P versus WT cells (Figure 3B,C; *p* < 0.0001). Our data suggest that imbalance between the generation of excess AP sites and inefficient repair in L22P mice stomach likely has the potential to lead to SSBs or may block DNA replication forks, resulting in DSBs. Previously, we provided evidence that L22P altered replication dynamics in human gastric epithelial cells [25]. To further determine whether the L22P mutation induces spontaneous DSBs in mice, stomach tissue sections were fixed and processed for immunohistochemistry using phosphorylated H2AX (γH2AX) as a DSB marker. We found that L22P mice exhibited a significantly increased frequency of DSBs compared with that of WT mice in the stomach tissue (Figure 3D,E; *p* < 0.001). Further, western blot analysis shows that γH2AX protein level was significantly higher in the L22P stomach than in the WT stomach (Figure 3F). To determine whether replication stress contributes to the production of DSBs in the L22P mouse stomach, we examined the Chk1 activation in the L22P stomach. We found that p-Chk1 (Ser317) activation was significantly increased in L22P stomach tissues (Figure 3G), suggesting that replication stress in L22P mice is a factor contributing to spontaneous DSBs. To examine whether exposure of alkylating agents (MMS) leads to accumulation of DSBs in L22P MEFs cells, we assayed γH2AX localization on WT and L22P MEFs cells by immunofluorescence. We stained cells with antibodies recognizing 53BP1 and γH2AX. Our data show that γH2AX and 53BP1 colocalization significantly increased in L22P MEFs versus WT MEFs (Figure 3H) suggesting that the number of DSBs in L22P MEFs cells accumulate up to six hours after MMS treatment (Figure 3H). In contrast, the percentage of γH2AX-positive cells was significantly decreased in WT cells after twelve hours of recovery from MMS treatment (Figure 3I). Our data suggest that the number of DSBs is significantly increased in L22P cells during alkylating DNA damage. 

### 2.4. L22P Causes Tetraploidy and Aneuploidy

**Figure 4:**
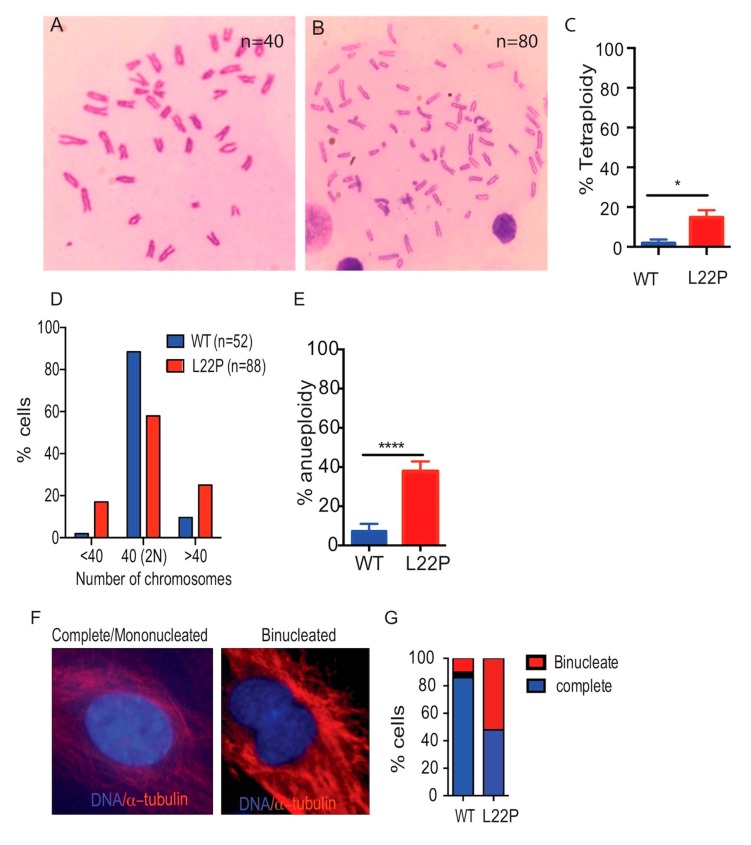
L22P cells cause abnormal number of chromosomes. (**A**) Representative image of diploid mouse embryonic fibroblast (MEF) chromosome spread with 40 chromosomes; (**B**) Tetraploid L22P MEFs cells chromosome spread with 80 chromosomes, All metaphase chromosome spread image were captured using 40× objective with bright field microscope; (**C**) Percent of tetraploid MEFs; (**D**) Histogram of chromosome spreads to show the spread of aneuploidy; (**E**) Average aneuploidy in MEFs (3 experiments of 50 spreads each; (**F**) The image of cytokinesis during cell division in WT and L22P cells indicates mononucleated versus binucleated cells, respectively. All image captured wit 63x objective of Zeiss fluorescence microscope (Zeiss, San Diego, CA, USA); (**G**) Percent of cells with binucleate formation, cells that fail to complete cytokinesis, and cells with complete cytokinesis for WT (*n* = 137) and L22P (*n* = 65) cells. All statistical analyses were carried out with Student’s *t*-test, and statistical significance is designated by * *p* < 0.05; **** *p* < 0.0001. All data were analyzed using GraphPad Prism software (Prism 7.00 for Mac, GraphPad Software, San Diego, CA, USA).

To determine whether L22P causes abnormal chromosome numbers, MEFs cells were prepared from E13.5 L22P embryos and WT littermates. L22P caused a significant increase in tetraploidy as assessed by the number of chromosomes doubled in metaphase spreads (Figure 4A,B). Although most of the WT cells had a diploid chromosome number (*n* = 40), L22P-expressing MEFs exhibited a 15% increase in tetraploidy (number of chromosomes = 80) versus WT MEFs (number of chromosomes = 40) (Figure 4B,C, *p* < 0.05). Furthermore, our data show that 40% of L22P-expressing MEFs cells showed aneuploidy that exhibited gain or loss of chromosomes followed by chromosomal mis-segregation (Figure 4D,E; *p* < 0.0001). Consistent with this, we further examined whether cytokinesis failure in L22P cells contributes to tetraploidy or leads to gain or loss of chromosomes. We found that the number of binucleated cells significantly increased in L22P versus WT cells (Figure 4F–G; *p* < 0.001), suggesting that Pol β may prevent cytokinesis failure tetraploidy and chromosomal segregation defects that lead to aneuploidy.

### 2.5. L22P Causes Mitotic Defects

**Figure 5:**
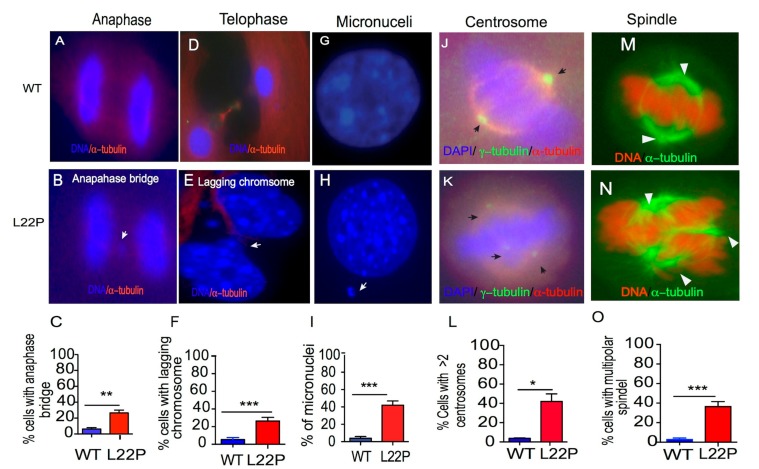
L22P cells exhibit mitotic defects. (**A**,**B**) Representative images of wild type (WT) and L22P cells with the anaphase bridge chromosomal segregation pattern; (**C**) Percent of cells with an anaphase bridge for WT (*n* = 62) and L22P (*n* = 57) cells; (**D**,**E**) Image of the lagging chromosomal structure in WT and L22P cells; (**F**) Percent of cells with lagging chromosomes at telophase for WT (*n* = 110) and L22P (*n* = 110) cells; the white arrow indicates lagging chromosomes; (**G**,**H**) Image of 4′,6-diamidino-2-phenylindole (DAPI)-stained nuclei from WT and micronuclei in L22P cells in H indicated with white arrow; (**I**) Percent of micronucleus-positive cells for L22P (*n* = 210) versus WT cells (*n* = 214). (**J**,**K**) Image of centrosomes stained for γ-tubulin and microtubules stained with α-tubulin antibody; the black arrow indicates the centrosomes; (**L**) Percent of cells with greater than two centrosomes for L22P (*n* = 168) versus WT (*n* = 120) cells; (**M**,**N**) Image of the assembly of cell poles stained for α-tubulin; the white arrow indicates two poles in WT versus the multiple assembly of tubulin in L22P cells; (**O**) Percent of cells with multipolar spindles >2 for WT (*n* = 109) and L22P (*n* = 87). All statistical analyses were performed with Student’s *t*-test and significance is represented by * *p* < 0.05; ** *p* < 0.01; *** *p* < 0.001 using GraphPad Prism (Prism 7.00 for Mac, GraphPad Software, San Diego, CA, USA). All images (A–N) were captured wit 63× objective of Zeiss fluorescence microscope (Zeiss, San Diego, CA, USA).

Previously, we have shown that L22P expression in gastric epithelial cells promotes chromosomal aberrations [25]. Here, we asked whether L22P MEFs cells are vulnerable to chromosomal segregation defects in mitotic cells. We quantified the number of cells with chromosomes that lag behind during anaphase or telophase, which are commonly used as markers of chromosome mis-segregation. We found that the percentage of cells with lagging chromosomes during anaphase and telophase significantly increased in L22P cells versus WT cells (Figure 5A–C; *p* < 0.01; Figure 5D–F; *p* < 0.001). Subsequently, we found that 45% of L22P cells harbor micronuclei compared to 3% of WT cells (Figure 5G–I; *p* < 0.001). Further, we hypothesized that chromosomal segregation defects observed in L22P cells may be a consequence of abnormal spindle formation. We examined spindle pole organization of mitotic cells by immunostaining using anti-γ-tubulin antibodies. Forty percent of L22P mitotic cells exhibit greater than two centrosomes (Figure 5J–L, *p* < 0.05). In addition, L22P MEFs cells exhibited multiple poles (Figure 5M–O; *p* < 0.001). These results provide further evidence supporting the notion that extra centrosomes contribute to assemble multipolar spindles, resulting in multipolar cell division. 

### 2.6. L22P Mice Exhibit an Inflammatory Response and Develop Tumors

**Figure 6:**
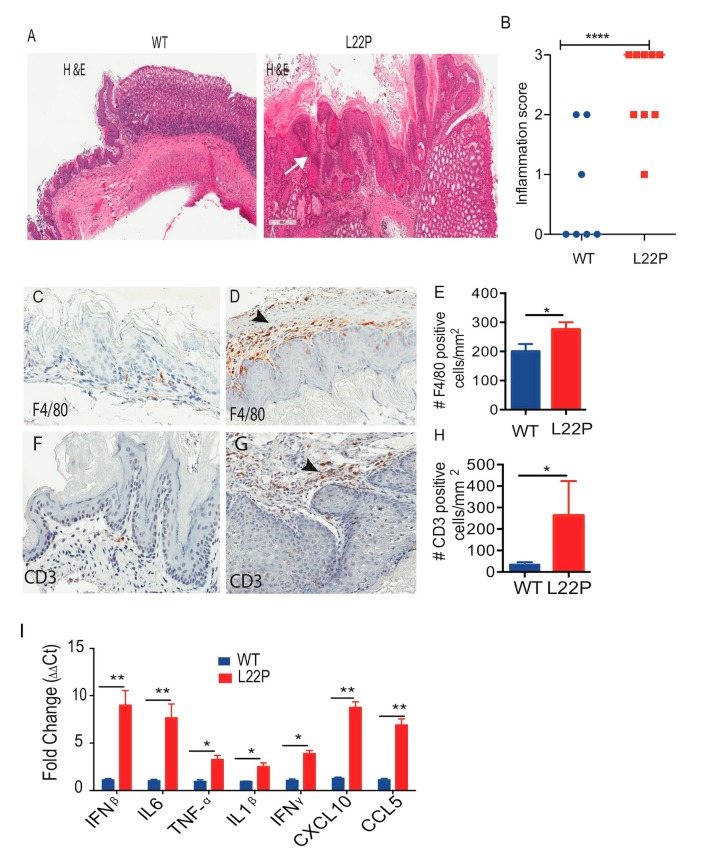
L22P increases susceptibility to gastric inflammation in mice. (**A**) Representative image of gastric histopathology of wild type (WT) and L22P C57Bl/6 mice with H and E staining; (**B**) Quantification of inflammatory score between WT (seven mice) and L22P mice (nine mice) stomach tissue sections; Low-to-medium-grade inflammation with abundant lymphocytes and neutrophilic granulocytes in L22P mice. Note that inflammation scores for forestomach and glandular regions are combined and white arrows (**A**) show pathological severity and infiltrating cells on forestomach and glandular regions; Note that inflammation score was done by grading pathological severity as normal (0), mild (1), moderate (2), severe (3); Images at Figure A and B shows 5× magnification and were captured using Scanscope (Leica Biosystem, Vista, CA, USA); (**C**,**D**) Macrophages stained with antibody against F4/80 in WT and L22P stomach tissue section; (**E**) Estimated number of macrophages/mm^2^ of tissues; (**F**,**G**) Lymphocytes infiltration (T-cells) stained with antibody against CD3 from WT and L22P mice stomach; (**H**) Quantification of the number of CD3 positive cells/mm^2^ of tissue; Images of the stomach tissue sections at C,D, F and G shows 10x and were captured using Scanscope (Leica Biosystem, Vista, CA, USA) (**I**) Upregulation of the mRNA expression of inflammatory cytokines (interferon gamma (IFNγ), interleukin (IL)-1β, tumor necrosis factor-alpha (TNFα), IL6, interferon beta (IFNβ)) and chemokines (C-X-C motif chemokine 10 (CXCL10), chemokine 5(CCL5)) significantly increased in L22P versus WT mice. Black arrows show infiltrating cells. All statistical data analyses were processed using GraphPad prism (Prism 7.00 for Mac, GraphPad Software, San Diego, CA, USA). Statistical significance is represented by * *p* < 0.05; ** *p* < 0.01; **** *p* < 0.0001.

**Figure 7:**
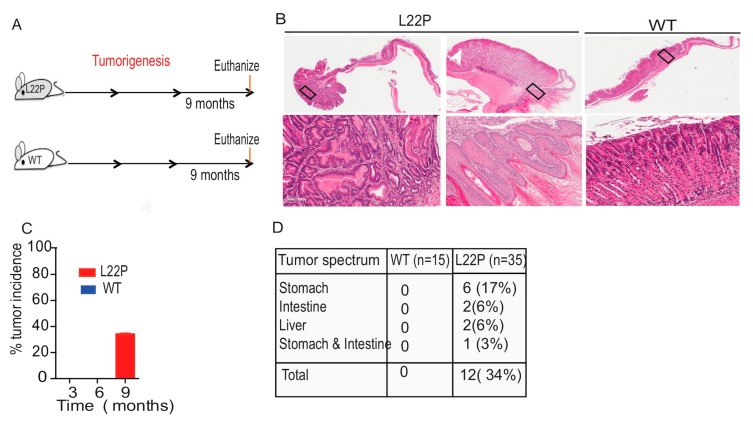
L22P increased tumor incidence and spectrum in mice. (**A**) Schematic representation of the tumorigenesis study’s experimental design (**B**) Representative tumor images wild type (WT) and L22P mice followed up for 9 months. Images of the stomach tissue sections shows 1× magnification (upper panel) and 10× magnification (lower panel) were captured using Scanscope (Leica Biosystem, Vista, CA, USA); (**C**) percent of tumor incidence in WT and L22P mice at 3 months (WT (*n* = 12); L22P (*n* = 13), 6 months (WT (*n* = 8); L22P (*n* = 18), and 9 months of experiment (WT (*n* = 15); L22P (*n* = 35); (**D**) Tumor spectrum was observed at 9 months of age.

To examine whether L22P mice exhibit inflammation in stomach tissue, histological analysis was performed on stomach tissues from 6-month-old mice using H and E staining (Figure 6A). We found that WT mice displayed moderate inflammation versus strong inflammatory changes in L22P mice at 9 months of age (Figure 6B). In addition, the immunostained gastric tissue from L22P mice shows infiltration of macrophages and lymphocytes (Figure 6C–H). Furthermore, infiltration of macrophages was higher in the stomach tissue of L22P versus WT mice (Figure 6D). The inflammatory infiltration, consisting of lymphocytes in the forestomach and the gastric glands, was present in severe cases to the submucosa layer (Figure 6F–H). Moreover, we asked whether inflammatory response in L22P mice stomach is linked to tumor development through the production of inflammatory cytokines. To examine whether L22P contributes to increased inflammatory cytokine and chemokine responses, we measured the mRNA expression levels of the tumor necrosis factor-alpha (TNF-α), interleukin-1beta (IL1-β), interferon beta (IFNβ), and IL-6 proinflammatory cytokines and chemokines (chemokine 5 (CCL5); C-X-C motif chemokine 10 (CXCL10)) using qRT-PCR and found a significantly increased level in L22P versus WT mice (Figure 6I). Our data shows that L22P mice have an amplified proinflammatory response that could contribute to tumor development. To determine whether L22P leads to specific pathological tissue responses, we evaluated gastric histological change and tumor development in L22P mice at 3, 6, and 9 months of age (Figure 7A). Strikingly, stomach tissues exhibiting hyperplasia and dysplastic regions were consistently more prominent in L22P mice (Figure 7B). Interestingly, we found that tumor incidence increased by 34% L22P mice until 9 months of age. (Figure 7C). Moreover, our findings show that the tumor spectrum includes stomach, intestine, and liver tumors (Figure 7D). In particular, 17% of L22P mice developed stomach tumors. In contrast, no tumors were found in the WT littermates at up to 9 months of age (Figure 7B,C). Thus, our data suggest that the dRP lyase activity of Pol β is critical to suppress tumor development. 

## 3. Materials and Methods

### 3.1. Mouse Breeding and Expression of L22P

All animal breeding and experiments were conducted in accordance with the Guide for the Care and Use of Laboratory Animals. The protocol used was approved by the University of Texas at Austin Animal Care and Use Committee (Kidane, protocol number AUP2017-00068, approval date: 19th March 2019–9th April 2020). To express L22P, we obtained an *EIIa-Cre* transgenic mouse from Jackson Laboratory (stock # 003314) and crossed it with our L22P*^flox/flox^* mice. All primers used for genotyping of mice are listed in Table S1.

### 3.2. Immunohistochemical Analysis

Tissue samples were collected from WT and L22P mice, washed in Phosphate-buffered saline (PBS), and fixed in 10% neutral-buffered formalin solution (pH 7.4) overnight. Tissues were embedded in paraffin and serially sectioned by the MD Anderson Research Histology Core Facility. Immunostaining for Ki67 and γH2AX was performed using rabbit anti-Ki67 (Bethyl Laboratories, Montgomery, TX, USA IHC-00375-1) and rabbit anti-γH2AX (Cell Signaling, Danvers, MA, USA 2577), respectively. Samples were imaged at 40× magnification with bright field using Aperio Scanscope (Nikon Instruments Inc., Melville, NY, USA), Leica Biosystems (Buffalo Grove, IL, USA). Data for Ki67 and γH2AX were scored blindly using two independent lab individuals. To score Ki67 and γH2AX-positive cells counted using each tissue section, at least 10 fields were used, and the average percentage of Ki67-and γH2AX-positive cells in three mice for every condition was plotted. Immunohistochemical analysis was performed as described previously [29]. Histopathological scoring of gastric mucosa was performed as described previously [30,31]. 

### 3.3. Comet Assays

Single-cell gel electrophoretic comet assay was performed under alkali conditions to detect single strand breaks. Cells were collected and rinsed twice with ice-cold PBS; 2 × 10^4^/mL cells were combined with 1% LMAgarose at 40 °C at a ratio of 1:3 (vol/vol) and immediately pipetted onto slides. For cellular lysis, the slides were immersed in the lysis solution (Trevigen, Gaithersburg, MD, USA, 4250-010-01) overnight at 4 °C followed by washing in the rinse buffer (90 mM Tris buffer, 90 mM boric acid, 2mM Na_2_EDTA at pH 8.5) for 30 min with two repeats. The slides were then subjected to electrophoresis at 20 V (0.6 V/cm) for 25 min and stained with SYBR green for 30 min. All images were taken with FITC filter using a Zeiss fluorescence microscope (Zeiss, San Diego, CA, USA) and analyzed by Open Comet Assay using Image J application as described previously [32].

### 3.4. AP Site Assay

Genomic DNA was isolated using DNAzol genomic DNA isolation reagents that contains guanidine/detergent lysis buffer that we purchased from Invitrogen (Grand Island, NY, USA). The AP assay was conducted after the DNA was labeled with Aldehyde reactive probe (ARP). The AP sites assay kit (Colorimetric; Abcam, Cambridge, MA, USA) utilizes the ARP reagent that reacts specifically with an aldehyde group, which is the open ring form of the AP sites. After treating DNA-containing AP sites with ARP reagents, AP sites are tagged with biotin residues, which can be quantified using an avidin–biotin assay followed by a colorimetric detection. The kit provides the necessary reagents for convenient determination of abasic sites in purified DNA in 96-well plate format. The number of AP sites was measured and calculated based upon a standard curve generated using ARP standard DNA solutions as described previously (DNA Damage AP sites assay kit, Colorimetric, Cat # ab211154; Abcam).

### 3.5. Immunofluorescence Localization 

Cover slips (Thermo FIsher, Waltham, MA, USA, 154526) were seeded with 20,000 cells and cultured for 24 hours. MMS treatments (1.5 mM) were appropriately performed. Then, the cells were fixed with 3.5% formaldehyde or methanol: acetic acid (3:1 ratio) for 10 minutes. The cells were then permeabilized in PBS containing 0.5% Triton X-100 for 15 minutes at room temperature. Then, the cells were blocked with 3% BSA in PBS for 1.5 hours at room temperature. The primary antibody listed in Table S2 was diluted to a 1:100 concentration in blocking buffer and incubated with the cells overnight at 4 °C. On the following day, the cells were washed with PBS and then incubated with secondary antibody diluted to a 1:400 concentration in blocking buffer for 1.5 hours. The secondary antibodies include Fluorescein isothiocyanate (FITC)-conjugated anti-mouse antibody (Jackson Labs, Bar Harbor, ME, USA, 715-095-150), FITC-conjugated anti-rabbit antibody (Jackson Labs, 711-095-152), TRITC-conjugated anti-mouse antibody (Jackson Labs, 715-025-150), and tetramethylrhodamine isothiocyanate (TRITC)-conjugated anti-rabbit antibody (Jackson Labs, 711-025-152). Finally, the cells were washed with PBS and mounted with cover slips using mounting medium containing DAPI stain (Vector Laboratories, Burlingame, CA, H-1200). Images were captured using a Carl Zeiss microscope with an AxioCam MRc5 (Zeiss) color camera. Nuclei staining positive for γH2AX were scored double blind using a subjective scale based on the number of colocalized foci. Greater than five was considered positive.

### 3.6. Quantitative Real-Time PCR

Tissue samples were homogenized in Eppendorf tubes using a pestle on ice. RNA was extracted using 1 mL Trizol (Thermo Fisher, 15596026) reagent. cDNA was synthesized from 2 µg of RNA using High-Capacity cDNA Reverse Transcription Kit from Applied biosystems (Applied Biosystem, Foster City, CA, 4368814). For the amplification of the target genes, 100 ng of cDNA was used in the final reaction mixture of 20 µL, with 10 µL of iTaq^TM^ Universal 2× SYBR® green supermix (Bio-rad, Hercules, CA, 172-5121), 500 nM of forward and reverse Primers. The samples were run in a ViiA7 Real-Time PCR System (Applied Biosystems, Foster City, CA) in 384 well plates. The PCR conditions were: 95 °C for 10 min, followed by 40 cycles at 95 °C for 15 s and annealing temperature for 1 min. The relative expression of gene was calculated by 2^(−∆∆CT)^ method and GAPDH was used as an internal control. All primer sequences are listed in Table S1. 

### 3.7. Immunoblotting

Western blotting was performed using a nitrocellulose membrane (BioRad, 162-0112) and the membrane was incubated with primary antibody against the respective proteins as described in Table S2. After extensive washing in PBS containing 0.1% Tween-20, the membrane was incubated with a 1:3000 dilution of anti-mouse (GE Healthcare, Chicago, IL, USA, NXA931) or anti-rabbit (GE Healthcare, NA934V) secondary antibody conjugated to HRP for 1 hour at room temperature, then washed and developed with enhanced ECL substrate (Thermo Scientific, Waltham, MA, USA, 34080). The gel images were captured by ChemDoc XRS (Bio-Rad). All antibodies are listed in Table S2.

### 3.8. Statistical Analysis

All the reported data were evaluated in a pairwise manner, comparing wild-type versus L22P cells using GraphPad Prism (La Jolla, CA, USA). Error bars represent mean ± standard error of the mean (SEM)unless otherwise specified. Statistical significance was concluded at < 0.05.

## 4. Discussion

This study demonstrates that mutations in the dRP lyase domain of Pol β induce chromosomal instability and accelerate inflammation associated carcinogenesis in mice. We have previously revealed that Pol β mutation induces genomic instability and cellular transformation [19,33,34]. Here, we present data generated from a novel L22P mouse model to investigate the consequence of how loss of dRP lyase activity contributes to chromosomal instability and carcinogenesis. Our results presented here further show that Pol β is required to maintain chromosomal stability. Previous biochemical studies demonstrated that the L22P variant of Pol β altered the ability of the dRP lyase domain to fold properly and resulted in compromised catalytic efficiencies for nucleotide insertion, nucleotide binding, insufficient dRP lyase, and low polymerase activity [24,35]. The data from this work illustrate potential mechanisms whereby the dRP lyase mutant variant of Pol β induces toxic BER intermediates. L22P-insufficient dRP lyase and negligible polymerase activity may provoke an alternative long-patch BER pathway (LP-BER) to process the 5’dRP group. However, the L22P variant does not support BER to promote strand displacement repair synthesis to facilitate excision of the 5′-dRP residue by the Flap endonuclease 1 (FEN1) nuclease [36]. In addition, L22P protein conformation changes [24] may also affect the DNA protein complex (DPC) formation that is required to remove structurally distinct AP sites (2-deoxyribonolactone (dL)) via the LP-BER pathway [37,38]. Therefore, BER intermediates overwhelm the BER machinery in L22P-expressing cells and may override the G1 cell cycle to accumulate replication-mediated DSBs (Figure 3). This observation is supported by the Chk1 activation in L22P stomach cells and is consistent with our previous report showing that L22P cells promote S-phase-specific DSBs [25,39]. Our findings raise the remarkable possibility that L22P cells accumulate more DSBs and progress into mitosis that likely increases anaphase bridges. These data strongly suggest that the presence of anaphase bridges contributes to subsequent micronucleus formation in L22P cells that likely drives chromosomal instability [40,41,42]. In this situation, unstable dicentric chromosomes likely cause micronuclei that contain ether centric or acentric chromosome segments. It is possible to predict that the lagging of dicentric chromosomes in L22P-expressing cells may be lost to a micronucleus, or mis-segregation of the whole dicentric chromosome to one daughter cell and cause acentric micronuclei. In the future, more detailed studies combining molecular karyotyping with Fluorescence in situ hybridization (FISH) studies may allow us to uncover dicentric chromosome organization and centromere content in cells harboring L22P mutations.

Mitotic defects in cells contribute to aneuploidy that is associated with abnormal numbers of centrosomes, mitotic spindles, and kinetochore apparatuses to enhance chromosome mis-segregation [43,44]. Our data show that L22P cells exhibit abnormal microtubule assembly, which suggests that the absence of spindle pole checkpoints [45]. Remarkably, our results are consistent with previously published data that have shown that loss of BRCA1 or BRCA2 causes abnormal microtubule assembly and chromosomal segregation defects [46,47,48]. Further, our data show that L22P mutation causes centrosome amplification that has the ability to enhance chromosomal instability and may promote tumor development [49,50,51]. Our findings provide an explanation for the frequent observation of multiple centrosomes in cells with damaged DNA as a result of L22P mutation-associated spontaneous DNA damage of a prolonged S-phase or failure of cytokines (Figure 5) [52,53]. Our results are consistent with other studies that demonstrate how deregulation of Pol β induces centrosome amplification-mediated chromosomal instability [54]. In addition, these results suggest a potential mechanism for aneuploidy resulting from L22P-expressing cells that progress to mitosis with supernumerary centrosomes. Taking these points together, our current data are consistent with the interpretation that loss of BER repair function could leave unresolved DNA damage in the genome that exacerbates chromosomal segregation defects, which may give rise to chromosomal instability.

Nevertheless, both the previous study and our current findings illustrate the role of the dRP lyase domain in maintaining genome integrity [25]. However, it is unknown whether altered function of dRP lyase activity induced genomic instability attracts immune cells and accelerates inflammation-associated gastric cancer. Chronic inflammation contributes to gastric carcinogenesis [55]. As we characterized infiltration of innate immune cells in the L22P mouse stomach (Figure 6A,B), we found a significantly increased infiltration of macrophages, lymphocytes, and gene expression of proinflammatory cytokines, suggesting that Pol β likely plays a significant role in maintaining host immune response hemostasis (Figure 6C–I). Our findings may indicate that the presence of infiltrated immune cells in the L22P mouse stomach may contribute to a cancer-prone microenvironment. Tumor initiation and progression is a multistep process that requires genomic instability and a tumor promoting inflammatory microenvironment. Our data demonstrate that L22P mouse stomach exhibits gastric pathology including hyperplasia, dysplasia, and inflammatory response (Figure 6A,B). It is also intriguing to note that spontaneous tumorigenesis studies have shown that L22P mutation increases tumor incidence and spectrum. However, the overall tumor incidence in L22P is approximately 34% at the age of 9 months (Figure 7C). In addition, L22P mice had an increased incidence of other types of cancers, suggesting that L22P is not stomach-specific. Overall, our findings reveal that insufficient dRP lyase and low polymerase activity of the L22P variant of Pol β may synergistically contribute to chromosomal instability and inflammatory-mediated tumor development. 

## 5. Conclusions

Altogether, our data presented here provide important insights into the way that Pol β contributes to genomic instability and carcinogenesis in mice. An important outcome from our work presented here is that the identification of abnormal mitosis in L22P cells likely provides growth advantages to induce cellular transformation that promotes tumor development [54,56]. Our results suggest that aberrant Pol β induces chromosomal instability but also impacts the inflammatory response, which may represent a novel insight for cancer etiology. Overall, our spontaneous tumor development study provides a unique opportunity to integrate the molecular mechanism whereby Pol β mutation contributes to carcinogenesis. Moreover, our L22P mouse model will be useful for future studies to uncover the mechanisms underlying human carcinogenesis. 

## Supplementary Materials

The following are available online at https://www.mdpi.com/2072-6694/11/8/1160/s1, Figure S1: Mutation in POLB moderately increase apoptosis; Figure S2: Image of Western blots; Table S1: list of primers; Table S2: List of antibodies.
