# Supplementary material for: Mutation in DNA Polymerase Beta Causes Spontaneous Chromosomal Instability and Inflammation-Associated Carcinogenesis in Mice
Source: Cancers (Basel). 2019 Aug 13;11(8):1160. doi: 10.3390/cancers11081160 (PMC6721533; doi:10.3390/cancers11081160)
Supplement: Supplementary file 1 [file cancers-11-01160-s001.zip › cancers-538111-supplementary.doc]

**Supplementary materials**

**Figure 1.** L22P modestly increased apoptosis in the glandular part of the stomach**.** (**A**,**B**) Image of the forestomach tissue section stained with anti-cleaved caspase-3 antibody to detect apoptotic cells in L22P mice; (**C**) Estimated percent of cells positive for cleaved caspase-3 in the forestomach (WT = 1970 cells; L22P = 1745 cells); (**D**,**E**) Glandular stomach area stained with cleaved caspase-3; All images (A,B,D,E) shows 10× magnification and were captured using Scanscope (Leica Biosystem, Vista, CA, USA). (**F**) Estimated percent of positive cells (WT = 4776 cells; L22P = 4407 cells). Note that three mice from each of the WT and L22P genotype groups were included to estimate the difference. All statistical analyses were performed using GraphPad Prism. *******p* <0.01 represent statistical significance and ns represent not statistical significance difference.

**Table S1.** List of primer sequences used for genotyping mice and RT-PCR.

| **Gene** | **Forward (5’-3’)** | **Reverse (5’-3’)** |
| --- | --- | --- |
| mCCL5 | TGCAGAGGACTCTGAGACAGC | GAGTGGTGTCCGAGCCATA |
| mIFN-ß | AGAAAGGACGAACATTCGGAAA | TCCGTCATCTCCATAGGGATCTT |
| mTNF-α | GTAGCCCACGTCGTAGCAAA | ACAAGGTACAACCCATCGGC |
| mGAPDH | TGCACCACCAACTGCTTAG | GGATGCAGGGATGATGTTC |
| mIFN-γ | CCAGGACCCATATGTAAAAGAAGC | TCATGTCTTCCTTGATGGTCTCC |
| mIL-6 | ACAAAGCCAGAGTCCTTCAGAGA | CTGTTAGGAGAGCATTGGAAATTG |
| mCXCL-10 | GGATGGCTGTCCTAGCTCTG | TGAGCTAGGGAGGACAAGGA |
| EIIa-Cre | CTAGGCCACAGAATTGAAAGATCT  GCGGTCTGGCAGTAAAAACTATC | GTAGGTGGAAATTCTAGCATCATCC  GTGAAACAGCATTGCTGTCACTT |
| Flox | AAGGACGGAAGGTGGAGGGAGAGCTAATGC | CGGCTTTCCATCCCTGCTAAGTGACAGC |
| L22P | AGCCGACGGTTGGCAT  ACACCTCCCCCTGAAC | CTGTACGCATTGTACTTGTGG |
| FP1/RP3 | CTCCTGGGCAACGTGCTG | GCGTGTGGCCTCGAACACC |
| FP2/RP4 | GGTGTTCGAGGCCACACG | GGTCTCCTGCGGCGCCTTG |
| FP5/RP7 | GAGCAAACGCAAGGCGCC | CCCCAGATGACTCCTATCCTC |
| Fprobe/Rprobe | GGCAGGCCCTCCGAGCG | CATCAAGGAAACCCTGGACTAC |
| L22P-sequencing | GTCTCACCCTTTGACAGG | |
| Gene | Forward (5’-3’) | Reverse (5’-3’) |
| mCCL5 | TGCAGAGGACTCTGAGACAGC | GAGTGGTGTCCGAGCCATA |
| mIFN-ß | AGAAAGGACGAACATTCGGAAA | TCCGTCATCTCCATAGGGATCTT |
| mTNF-α | GTAGCCCACGTCGTAGCAAA | ACAAGGTACAACCCATCGGC |
| mGAPDH | TGCACCACCAACTGCTTAG | GGATGCAGGGATGATGTTC |
| mIFN-γ | CCAGGACCCATATGTAAAAGAAGC | TCATGTCTTCCTTGATGGTCTCC |
| mIL-6 | ACAAAGCCAGAGTCCTTCAGAGA | CTGTTAGGAGAGCATTGGAAATTG |
| mCXCL-10 | GGATGGCTGTCCTAGCTCTG | TGAGCTAGGGAGGACAAGGA |
| EIIa-Cre | CTAGGCCACAGAATTGAAAGATCT  GCGGTCTGGCAGTAAAAACTATC | GTAGGTGGAAATTCTAGCATCATCC  GTGAAACAGCATTGCTGTCACTT |
| Flox | AAGGACGGAAGGTGGAGGGAGAGCTAATGC | CGGCTTTCCATCCCTGCTAAGTGACAGC |
| L22P | AGCCGACGGTTGGCAT  ACACCTCCCCCTGAAC | CTGTACGCATTGTACTTGTGG |
| FP1/RP3 | CTCCTGGGCAACGTGCTG | GCGTGTGGCCTCGAACACC |
| FP2/RP4 | GGTGTTCGAGGCCACACG | GGTCTCCTGCGGCGCCTTG |
| FP5/RP7 | GAGCAAACGCAAGGCGCC | CCCCAGATGACTCCTATCCTC |
| Fprobe/Rprobe | GGCAGGCCCTCCGAGCG | CATCAAGGAAACCCTGGACTAC |
| L22P-sequencing | GTCTCACCCTTTGACAGG | |

**Table S2.** List of antibodies used for this study.

| **Antibody** | **Company** | **Dilution** |
| --- | --- | --- |
| anti-α-Tubulin | Santa Cruz (sc-5386) | 1: 500 |
| anti-Rad51 | Santa Cruz (sc-8349) | 1: 500 |
| anti-γH2AX | Millipore (07-164) | 1: 750 |
| anti-Chk1 | Cell Signaling (2360S) | 1: 1000 |
| anti-p-Chk1 | Cell Signaling (4947) | 1: 500 |
| anti-53BP1 | Santa Cruz (sz22760) | 1:200 |
| HRP-anti-Rabbit | GE healthcare (NXA934) | 1: 3000 |
| HRP-anti-Mouse | GE healthcare (NXA931) | 1: 3000 |
